# Integrated Platform for Monitoring Single-cell MAPK Kinetics in Computer-controlled Temporal Stimulations

**DOI:** 10.1038/s41598-018-28873-1

**Published:** 2018-07-24

**Authors:** Hyunryul Ryu, Minhwan Chung, Jiyoung Song, Sung Sik Lee, Olivier Pertz, Noo Li Jeon

**Affiliations:** 1 Research Laboratory of Electronics, Massachusetts Institute of Technology, 77 Massachusetts Avenue, Cambridge, MA 02139 USA; 2 Department of Mechanical Engineering, Seoul National University, Seoul, 151-742 Republic of Korea; 3 ScopeM (Scientific Center of Optical and Eletron Microscopy), ETH Zurich, Otto-Stern-Weg 3, CH-8093 Zurich, Switzerland; 4 Institute of Cell Biology, University of Bern, Baltzerstrasse 4, 3012 Bern, Switzerland; 5 Institute of Bioengineering, Seoul National University, Seoul, 151-742 Republic of Korea

## Abstract

Extracellular response kinase (ERK) is one of the key regulator of cell fate, such as proliferation, differentiation and cell migration. Here, we propose a novel experimental pipeline to learn ERK kinetics by temporal growth factor (GF) stimulation. High signal-to-noise ratio of genetically encoded Fluorescence resonance energy transfer (FRET) biosensor enables to get a large number of single-cell ERK activity at each time point, while computer-controlled microfluidics fine-tune the temporal stimulation. Using this platform, we observed that static Epidermal growth factor (EGF) stimulation led to transient ERK activation with a significant cell-to-cell variation, while dynamic stimulation of 3′ EGF pulse led to faster adaptation kinetics with no discrepancy. Multiple EGF pulses retriggered ERK activity with respect to frequency of stimulation. We also observed oscillation of ERK activity of each cell at basal state. Introducing of Mitogen-activated protein kinase kinase (MEK) inhibitor, U0126, was not only dropping the average of basal activity for 7.5%, but also diminishing oscillatory behavior. Activity level raised up when inhibitor was removed, followed by transient peak of ERK kinetics. We expect this platform to probe Mitogen-associated protein kinase (MAPK) signaling network for systems biology research at single cellular level.

## Introduction

Mitogen-associated protein kinases (MAPKs) are the key molecules delivering changes from the outside to cellular components^1^. Ras-MAPK pathway has been known as a central player in the development and progression of cancer^2^. Localized MAPK in the nucleus works as an initiator of transcriptional response^3,4^. Not only the presence of the molecule, kinetics of the molecule activity is playing an central role to determine the biological outcome^5–8^. Rat pheochromocytoma cell line, PC-12, is well-studied that sustained activity of ERK by neuronal growth factor (NGF) stimulation leads cells to be differentiated, while transient activation kinetics from epidermal growth factor (EGF) induces proliferation^5,9,10^. Systems biology have enlightened the mathematical links between molecules, explaining these dynamic responses, and predicting cellular behaviors^11–13^. However, average-based analysis method have been issued recently. Only the common kinetics of bio-molecule have been studied, disregarding the diversity of reactions^14^. Stem cell, for example, is likely to to differentiate to designated cell type, with a minor number of unwanted cell, which is capable of deteriorating the entire stem cell theraphy. Study of the single cell kinetics can enrich the knowledge about circuit structure and function of the signaling pathway which could not be otherwise revealed^14^.

Recently, there have been a significant breakthrough; genetically-encoded biosensor. Shankaran *et al*. introduced ERK-GFP fusion protein and analyzed periodic response of ERK at single cellular level^15^. Harvey *et al*. presented genetically encoded FRET-based sensor for ERK repsonse observation^16^. Fritz *et al*. proposed sensitive FRET-based biosensor with high signal-to-noise ratio which can be observed with 20x air objectives with less light^17^. By observing the individual kinetics in a real-time, theoretically suggested characteristics were verified, such as basal ERK oscillation^18,19^. A variety of MAPK responses have been analyzed and compared to different stimulation experiments. Harvey *et al*. and Fritz *et al*. showed ERK response by EGF stimulation^16^. Purvis *et al*. showed p53 dynamics at damaged cell by gamma-irradiation^6^. Selimkhanov *et al*. reported complex kinetics of ERK, calcium and NF-κB by EGF, ATP and LPS^20^. However, these methods were limited to static stimuli, which acts as a limiting factor in the analysis of the system properties of the molecular pathways. Precisely controlled temporal stimulation is to overcome these limitations, by providing a quantitative input, giving not only the mathematical characteristics about the pathway, but also enabled dynamic regulation of the gene expression^21,22^.

Here, biosensor and microfluidics, were integrated to observe ERK response from the pre-defined stimulation in a real-time. EKAR2G, FRET-based ERK biosensor, was genetically encoded, providing stable and uniform expression throughout the population. Meanwhile, the medium-filled reservoirs of microfluidic device were pressurized with computer-controlled pressure pump to manipulate GF concentration in the cell chamber. By measuring the intensity of ratio-metric single cell images, we analyzed the discrepancy of individual ERK kinetics to various stimulation patterns; sustained, pulsed and multi-pulsed. Basal oscillation of ERK activity of each cell was observed. Presenting of MEK inhibitor, U0126, was able to drop the average of basal activity and also eliminate amplitude of oscillation. Taking advantage of precise control of the stimulation regimes and high-throughput imaging capability with air objectives, we expect this integrated platform to be used to obtain quantitative data, establishing advanced mathematical models of MAPK dynamics.

## Experimental Section

### Generation of sensor-expressing cell-line and cell culture

HEK293T and HEK293 β5 cells (American Type Culture Collection) were cultured in Dulbecco’s modified Eagle’s medium (DMEM) supplemented with 10% fetal bovine serum (FBS), 1% L-glutamine, and 1% penicillin/streptomycin. We produced lenti-viral vectors expressing EKAR2G1 to establish stable cell lines, as used in previous research^17^. Briefly, HEK293T cells (Invitrogen, USA) were transfected with lenti-virus and packaging constructs. Supernatant was then collected and concentrated with a Lenti-X concentrator kit (Clontech, Japan). HEK293 β5 cells were infected and selectively cultured with 1 µg/ml puromycin (Sigma, Germany). Then, cells were sorted by flow cytometry to express homogeneous and high level of biosensor. After sorting, cells were subsequently cultured in presence of 1 µg/ml puromycin. At 70% confluence, cells were passaged gently with 0.25% Trypsin-EDTA (Sigma, Germany). Note that EKAR2G measures ERK phosphorylation activity specifically in the cytosol. This readout might therefore slightly differ from pERK antibody staining that measures the absolute amount of phosphorylated ERK.

### Design and fabrication of microfluidic device

For mammalian cell experiment, microfluidic device was modified from the previous research^23^. Previously, device was designed to stimulate *Saccharomyces cerevisiae* for systems biology research. The height of cell culture chamber was 40 µm. However, for mammalian cell with 20 µm diameter, this was not appropriate to provide healthy micro-environment. On the other hand, the higher height of the device the more medium between control part and cell chamber, which cause time delay on temporal stimulation. We separate the microfluidic device into two part with two-layered system; 40 µm for micro-channels of control part and 100 µm for cell chamber region. Cells were stabled within cell culture region, while cellular environment switched within 30 seconds between on-and-off states.

Microfluidic device was replicated from a Silicon wafer with SU-8 micro-structures. Silicon master mold was composed of 40 µm and 100 µm thickness layers of photoresist (PR). First, the plasma treated Silicon wafer was spin-coated with SU-8 100 (Microchem, USA) negative PR for 40 μm thick. After baking at 65 °C for 5 minutes and 95 °C for 20 minutes, wafer was masked by the negative film mask (Han & All Tech, Korea), and exposed to 250 mJ of 405 nm ultraviolet light. (Shinu MST, Korea) Wafer was, then, baked again at 65 °C for a minute and 95 °C for 10 minutes. SU-8 developer (Microchem, USA) was used to remove unexposed part. The second layer of PR was spin-coated for 100 μm thick, and baked at 65 °C for 10 minutes and 95 °C for 30 minutes. Film mask for the second layer was aligned using alignment pattern on the first developed layer. Wafer was exposed to 500 mJ of UV light. After the baking step at 65 °C for a minute and 95 °C for 10 minutes, wafer was dipped into the developer, and baked to evaporate the residual solvents on the top.

Poly-dimethylsilosane (PDMS) was used to replicate the master. Elastomer base and curing agent (Sylgard 184, Dow Corning) was mixed at a 10:1 ratio and degassed in a vacuum chamber for 5 minutes. Precursor was poured on the top of Silicon mold for 7 grams, and solidified at 80 °C for 30 minutes. Plastic reservoirs from 8-well strip (Evergreen sci, USA) were glued with precursor. Additional 30 g of precursor was poured to seal reservoirs. The replica was cut and punched as shown in Fig. S1A. PDMS replica and coverslip (Tasumi, Japan) were plasma treated and bonded irreversibly. To enhance the bonding strength, device was heated for 5 minutes on 80 °C hot plate. Microfluidic device was immediately filled with PBS to avoid bubble trapping.

### Preparation of microfluidic device and cell seeding

Prior to cell culture, microfluidic device was coated with poly-D-lysine (PDL, Sigma, Germany). Reservoirs on control part was filled with 2 μg/ml of PDL solution and kept in room temperature for at least 6 hours. PDL solution was washed out before cell culture. HEK293 β5/EKAR2G1 cell-line were prepared with concentration of 2 × 10^6^ cells/ml. Outlet reservoir connected to cell chamber was filled with 50 μl of suspension. Cells were flowing toward the cell chamber by hydrostatic pressure. After 30 minutes incubation, residual cells in the outlet were removed and replenished with fresh medium.

### Live cell imaging and monitoring of ERK biosensor

All experiments were performed on an Eclipse Ti inverted fluorescence microscope (Nikon, Japan) with Plan Apo air 20x (NA: 0.75) objectives controlled by Metamorph. (Molecular Devices, USA) Hamamatsu Orca R2 camera was used to acquire images at a 16-bit depth. Donor and FRET images were acquired sequentially using motorized filter wheels with the following excitation, dichroic mirrors, and emission filters (Chroma, USA): donor channel: 430/24×, Q465LP, 480/40 m; FRET channel: 430/24×, Q465LP, 535/30 m; mCherry channel: ET572/35, 89006bs, 632/60 m for dextran imaging. Standard exposition settings were used throughout the experiments. 440 nm, donor and FRET channel excitation, and 565 nm, red channel, LED lamps were used as light sources (Lumencor, USA), with 1.1% for 440 nm and 1.5% for 565 nm of LED power. To minimize photo-damage, exposure time was 300 ms for donor channel and 300 ms for FRET at binning 2 × 2.

Before the experiment, medium in each device was changed into the starvation medium, DMEM with 0.2% FBS. Each reservoir was filled with GF-containing or starvation medium for up to 200 μl. Microfluidic device was placed on the stage of microscope which is covered and controlled with heating source to stabilize the temperature at 37 °C. To prevent evaporation during the experiment, cell inlet port was sealed with transparent adhesive tape. Custom-made syringe connector was connected to the device (Fig. S1A). Built-in software of ONIX pressure pump (Millipore, USA) was used to control the valve sequence and pressure. Valve pressure was consistently set to 1.5 psi. All the protocols for stimulation experiments included 80 minutes of flow adaptation time in starvation medium to stabilize the baseline of ERK activity (Fig. S2A). Changing the opening sequence of the pressure valve, stimulation profile was temporally controlled (Fig. S1B,C).

Ratio-metric analysis of each single cell was calculated with Metamorph and ImageJ. Donor and FRET images were background-subtracted image by image. Image from FRET channel divided by the one from donor channel, and multiplied by 1000 to produce a 16-bit ratio-metric image. Projected ratio-image of time series was used to segment each coordinate of single cell. Cell clumps were discarded. Emission ratio of each cell through time was measured. The average emission ratio of 5 time points around 80-minute time point was set as the basal ERK activity level. Temporal GF stimulation can be identified using Rhodamin-dextran obtained from the time series of mCherry channel.

## Results and Discussion

### Experimental setup

**Figure 1:**
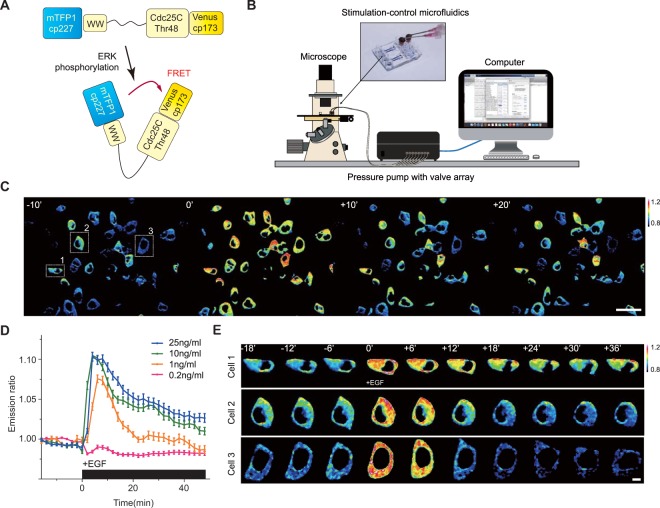
(**A**) Schematic of EKAR 2G1 FRET biosensor. It was stably expressed on HEK 293 cell-line. (**B**) Arrangement of Microfluidic device. Computer-controlled pressure pump was connected to the control part of the device, which manipulate temporal stimuli of the cell chamber. Fluorescent inverted microscope was used for live-cell monitoring. (**C**) Time series imaged using a 20x air objective. Stimulation introduced at 0 minute. Scale bar is 100 µm. (**D**) Average profile of ERK kinetics by different concentration of EGF. Stimuli introduced at 0′. (**B**) Three representative time series of cell from a single field of view. Each cell experienced ERK excitation by EGF stimulation, with diverse decaying kinetics. Scale bar is 20 µm.

**Figure 2:**
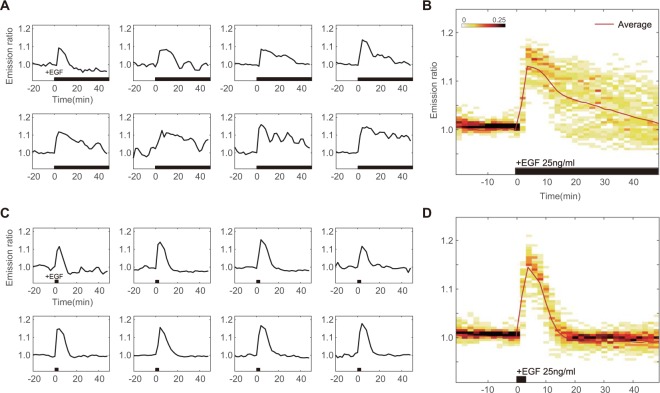
(**A**) Representative ERK trajectories from eight cells and (**B**) Cell density distribution of ERK activity in sustained stimulation experiment of 25 ng/ml EGF. Each curves spread over the average kinetics in wide range of ERK activity distribution. (**C**) Representative ERK trajectories from eight cells and (**D**) Cell density distribution of ERK response by pulsatile stimulation experiment of 25 ng/ml EGF. Temporally controlled stimulation induced synchronized ERK kinetics through the population. Black bar on time axis indicates timing of GF in.

FRET based ratio-metric biosensor have been a powerful tool for research on cell signaling dynamics. We used genetically encoded EKAR2G1 biosensor, which was verified previously (Fig. 1A)^17^. Biosensor was stably and homogeneously expressed in HEK293 cell-line. Meanwhile, microfluidic device was connected to computer-controlled pressure pump, and arranged to live-cell imaging system as shown in Fig. 1B. It could be stimulated precisely in temporal manner, by toggling the pressure valve on-and-off. As shown in Fig. S1A, control part was connected to each pressure valve (V1 and V2). Each reservoir of control part contains medium with or without GF. On-and-off state of cell chamber is controlled by switching V1 and V2 (Fig. S1B). Rhodamin-dextran have been introduced in GF medium to verify temporal stimulation. By toggling two state through time, we could dynamically control microenvironment of cell (Fig. S1C). To set the baseline of our platform, ERK activity was measured in microfluidic device without any GF medium (Fig. S2A). Immediate ERK excitation was detected when the connected reservoir was pressurized, and stabilized after 80 minutes. It was reported previously that shear stress was capable to activate MAPK signaling pathway^24^. Stimulation was taking place after at least 100 minutes to stabilize (Fig. S2B). ERK activity of the previous 80 minutes was considered irrelevant to GF stimulation. We used the average of emission ratio intensity for 5 time points around 80-minute to normalize each single cell kinetics. Due to high signal-to-noise ratio, a large number of single cell could be captured in each microscopic view (Fig. 2C). Transient ERK excitation was evoked immediately when EGF was introduced in the cell chamber (Fig. 1D). Ultra-sensitivity, which is well-known characteristics of ERK signaling pathway, was observed in our platform^25^. EGF stimulation of 1 ng/ml already gave 97.7% of saturated maximum peak intensity, while 0.2 ng/ml barely excite the pathway. Figure 1E shows three different single cell kinetics from the same field of view. ERK activity of Cell 1 sustained longer than 30′ after stimulation, while Cell 2 and 3 experienced adaptive ERK kinetics with different decaying time. This result suggest that average response analysis might miss these minor, but significant number of cell which give considerably varied behavior.

### ERK dynamics by temporally defined stimulus

From 60 curves of sustained 25 ng/ml EGF stimulation experiment, we randomly choose 8 kinetics (Fig. 2A). In average, which might be resulted by usual biochemical method, only transient excitation kinetics could be observed. However, as shown in Fig. 2A, the single cell behavior clearly varied from cell to cell. Figure 2B showed ERK activity distribution for each time point by sustained EGF stimulation. Maximum emission ratio was shown insignificant difference amongst cells. However, cells were gradually wider the range of ERK activity distribution.

Taking advantage of computer controlled microfluidic-pump system, we looked at how temporally-varied stimulation patterns alter the ERK dynamics. EGF stimulation was applied in pulsatile regime to the cell chamber. In contrast to sustained EGF stimulation, we observed the immediate de-activation of ERK. As shown in Fig. 2C, randomly chosen curves were shown no significant difference in 3′ pulsed stimulation, compare to sustained stimulation experiment. Distribution of ERK kinetics was also remarkably coordinated (Fig. 2D). Pulsatile stimulation gave synchronized ERK kinetics throughout the population.

**Figure 3:**
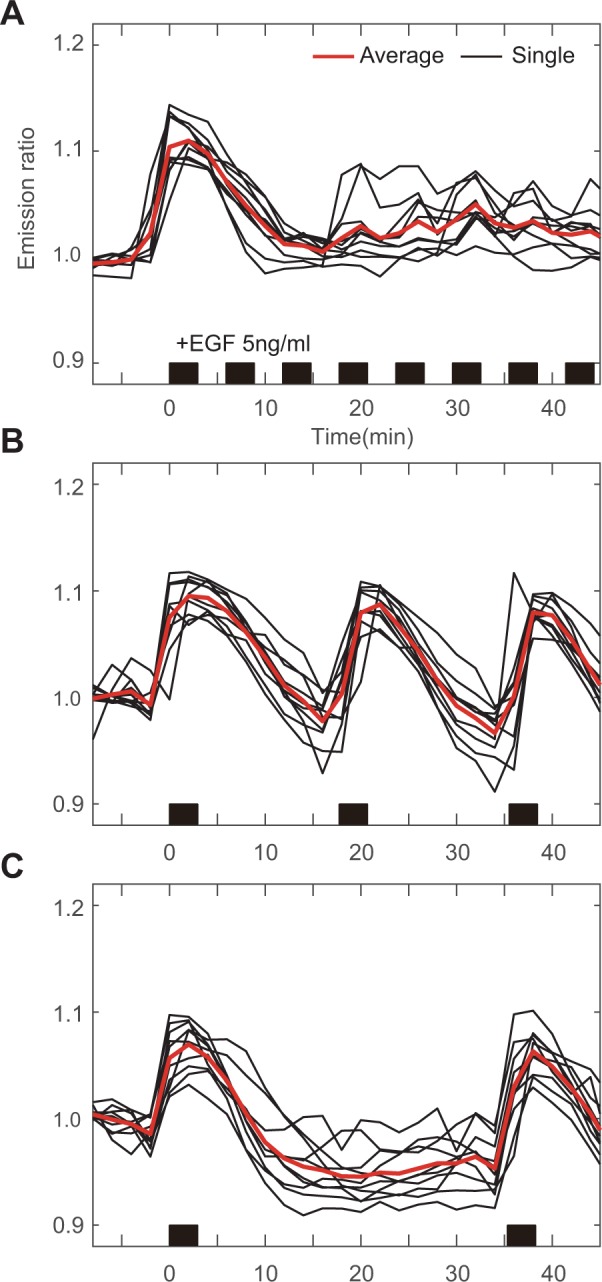
Average and single cell trajectories of ERK activity to frequent 3-minute pulsatile stimulation with interval of (**A**) 3 minutes, (**B**) 15 minutes, and (**C**) 33 minutes in 5 ng/ml EGF. Stimulation started at 0 minute. Black bar on time-axis represent the presence of GF at each time point.

ERK response to the frequent pulsatile stimulation was observed. High frequency stimulation was filtered out, giving transient excitation curves similar to sustained experiments (Fig. 3A). In contrast, with low frequency stimulus, ERK activity was triggered according to input timing (Fig. 3B and C). This result suggest that ERK signaling pathway show as low-pass filter to an external stimulus.

### ERK dynamics by temporally controlled MEK inhibitor

**Figure 4:**
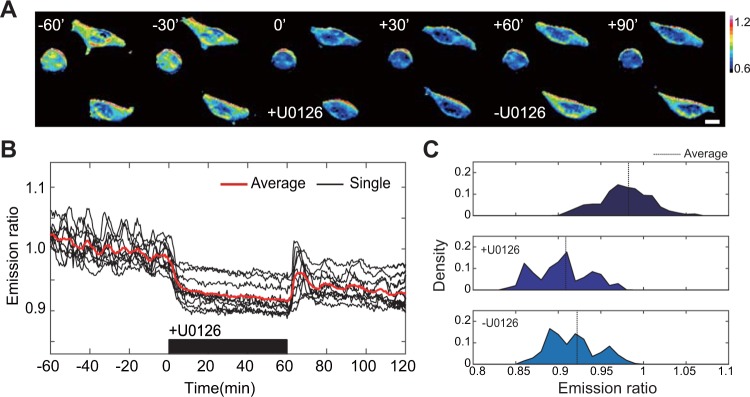
Basal ERK activity response to temporal inhibition of MEK. (**A**) Times series imaging. Scale bar is 20 µm. (**B**) Ratio-metric profile of ERK dynamics. Stimulation started at 0 minute. Black bar on time-axis indicate the presence of U0126. (**C**) Distribution of ERK activity at basal state (top), before (middle) and after (bottom) introducing 10 µM U0126.

Not only GF, pharmaceutical inhibitor could be introduced to cell chamber in temporal manner (Fig. 4A). In our previous study, it was limited to observe the detailed oscillatory behavior of ERK in PC-12 cell due to the relatively larger interval time, e.g. 2 minutes^26^. However, in this research, using HEK 293 β5 cell line, which is more flat and relatively bigger cell, we can obtain a higher sensitivity of the biosensor signal to lower the time between frames to 30 seconds. Figure 4B showed the basal level activity of the ERK captured in every 30 second. Basal ERK oscillation of ERK was observed as reported previously^15,19^. Since these oscillations cancel each other between cells, average plot could not capture this phenomena. At 0′, medium containing 10 µM of MEK inhibitor, U0126, was introduced into cell chamber. We treated the cells with MEK inhibitor, U0126, to validate that the fluctuation was coming from the cascade, itself, not the artifacts of the assay. Presenting of inhibitor was not only dropping the average of basal activity for ~7.5%, but also diminishing the oscillation itself (Fig. 4B). Additionally, we could monitor the ERK activity while microfluidic device washed out the inhibitor from cell chamber. Cells were transiently excited above the original basal level, and gradually recovered to the initial state. As shown in Fig. 4C, ERK activity distribution dropped in presence of U0126, and raised up after washing out.

## Conclusion

In this study, we integrated two well-characterized techniques; FRET biosensor and microfluidics. Highly sensitive and stable biosensor and computer-controlled microfluidics enable to monitor a individual ERK kinetics by various GF stimulation pattern in a high throughput manner. It was observed that sustained EGF stimulation induced a wide range of variation on ERK activities of each cell, while pulsatile stimulus synchronized kinetics over population. Low frequency of EGF pulse could retrigger the kinetics, however, high frequency stimulation was filtered out. Basal ERK oscillation was observed, and could be diminished by introducing MEK inhibitor. Removal of MEK inhibitor provoke immediate transient ERK excitation above the baseline, and gradually recover to original state. By observing a variety of ERK dynamics by temporal stimulation, we expect to gather quantitative data for single cell kinetics from different cell types, enhancing the knowledge about the pathway coordination. Exploring cellular responses by defined stimulation, this will produce ‘a common language’ that could lead a better interaction between experimentalists and theoreticians^27^.

## Electronic supplementary material

Supplementary Information

Movie S1

Movie S2

Movie S3

Movie S4
