# Supplementary material for: Integrated Platform for Monitoring Single-cell MAPK Kinetics in Computer-controlled Temporal Stimulations
Source: Sci Rep. 2018 Jul 24;8:11126. doi: 10.1038/s41598-018-28873-1 (PMC6057930; doi:10.1038/s41598-018-28873-1)
Supplement: Supplementary file 1 — Supplementary Information [file 41598_2018_28873_MOESM1_ESM.pdf]

Integrated Platform for Monitoring Single-cell MAPK Kinetics in Computer-controlled Temporal Stimulations

Hyunryul Ryu 1, Minhwan Chung 2, Jiyoung Song 2, Sung Sik Lee 3, Olivier Pertz 4, and Noo Li Jeon2,5*

1 Research Laboratory of Electronics, Massachusetts Institute of Technology, 77 Massachusetts Avenue, Cambridge, MA 02139, USA.

2 Department of Mechanical Engineering, Seoul National University, Seoul, 151-742, Republic of Korea

3 ScopeM (Scientific Center of Optical and Eletron Microscopy), ETH Zurich, Otto-Stern-Weg 3 CH-8093 Zurich, Switzerland

4 Institute of Cell Biology, University of Bern, Baltzerstrasse 4, 3012 Bern, Switzerland

5 Institute of Bioengineering, Seoul National University, Seoul, 151-742, Republic of Korea

*Correspondence to: njeon@snu.ac.kr

Keywords: Microfluidics, FRET biosensor, ERK dynamics, Single-cell kinetics.

Supplementary Information

Figure S1. Design of Microfluidic device. (A) Digital photograph of microfluidic device, bonded on 24 X 60 mm coverslip. (B) Schematic illustration of temporal stimulation. Switching on-and-off state provide pulsatile stimulation to cell chamber. (C) Intensity profiles of computer-controlled temporal stimulation.

Figure S2. (A) Average and single cell trajectories of ERK kinetics measured without any GF stimulation. Actual stimulation induced at least 80 minutes after monitoring to stabilize the measurement. (B) Average and single cell trajectories with 25ng/ml EGF stimulation. Stimulation started at 0 hour. Black bar on time-axis represent the presence of GF at each time point.

Figure S3. Average and single cell trajectories of ERK activity by various stimulation patterns. Stimulation started at 0 minute. Black bar on time-axis represent the presence of GF at each time point.

Movie S1. Demonstration of GF stimulation, visualized by Rhodamin-dextran. Scale bar is 100μm.

Movie S2. Time series of ERK response by sustained GF stimulation with 25 ng/ml EGF in a whole field of view by 20x objective (left), and 3 representative cell. (right) Scale bar is 20μm.

Movie S3. Time series of ERK response by 3-minute pulsatile GF stimulation with 25 ng/ml EGF in a whole field of view by 20x objective (left), and 3 representative cell. (right) Scale bar is 20μm.

Movie S4. Basal level ERK activity observed with 30-second time resolution. Scale bar is 20μm.

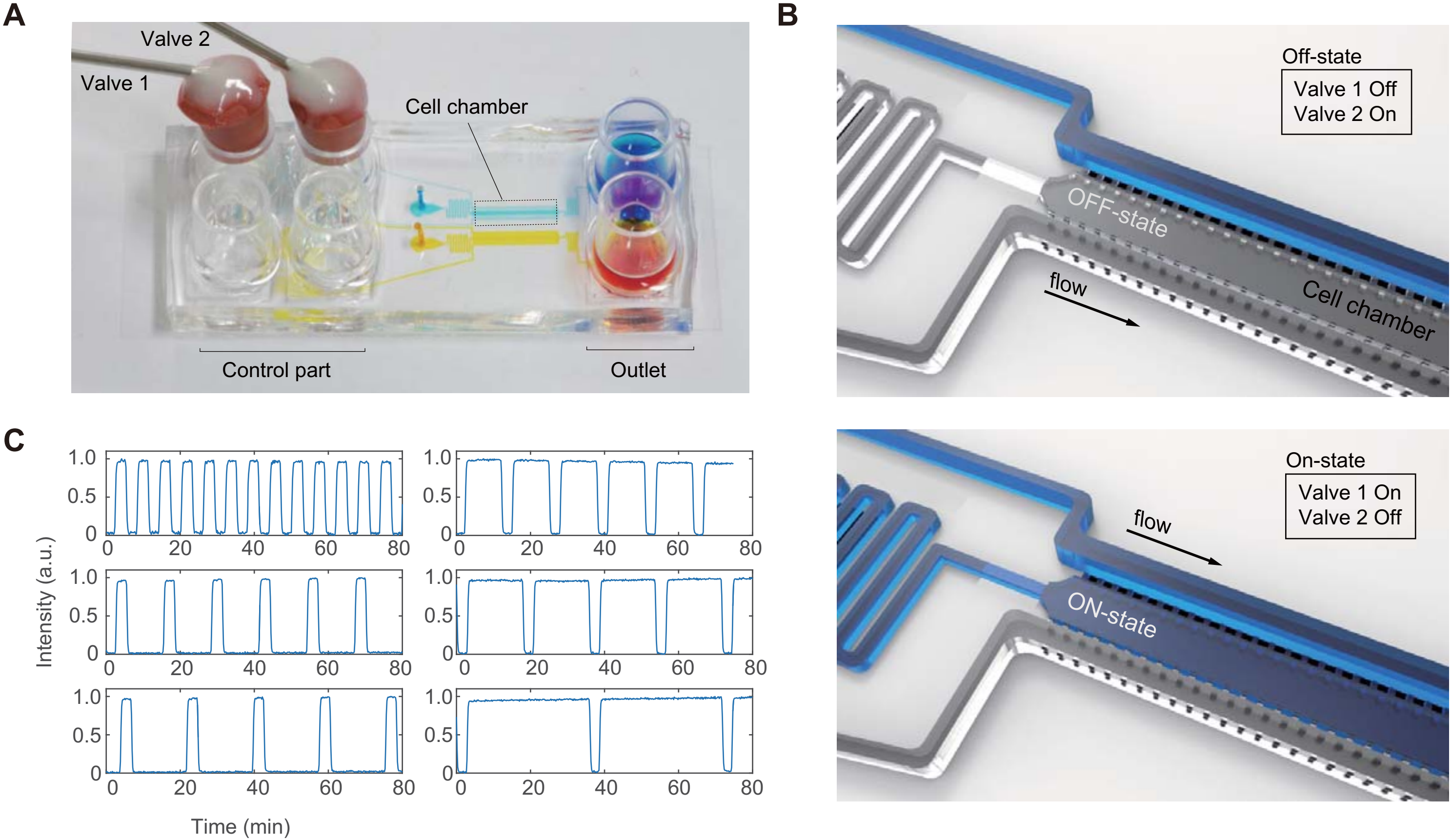
A
Valve 2
Valve 1
Cell chamber
Control part
Outlet
B
Off-state
Valve 1 Off
Valve 2 On
OFF-state
flow
Cell chamber
On-state
Valve 1 On
Valve 2 Off
flow
ON-state
C
Intensity (a.u.)
Time (min)
1.0
0.5
0
0
20
40
60
80

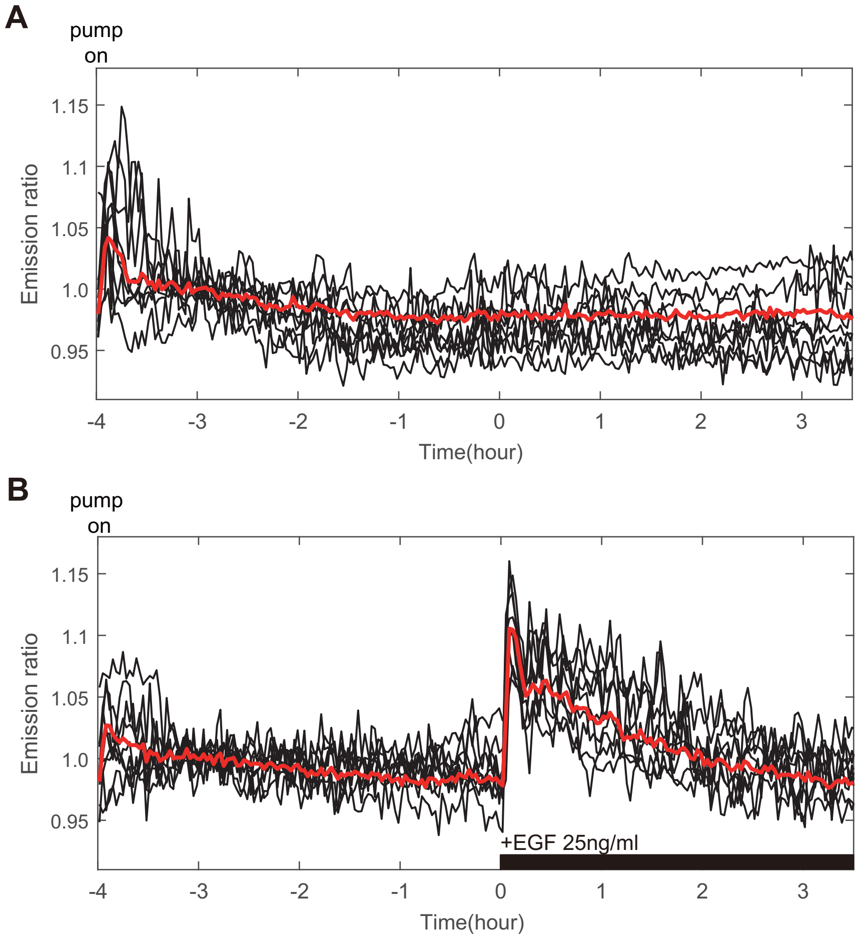
A
pump on
Emission ratio
1.15
1.1
1.05
1.0
0.95
-4
-3
-2
-1
0
1
2
3
Time(hour)
B
pump on
Emission ratio
1.15
1.1
1.05
1.0
0.95
+EGF 25ng/ml
-4
-3
-2
-1
0
1
2
3
Time(hour)

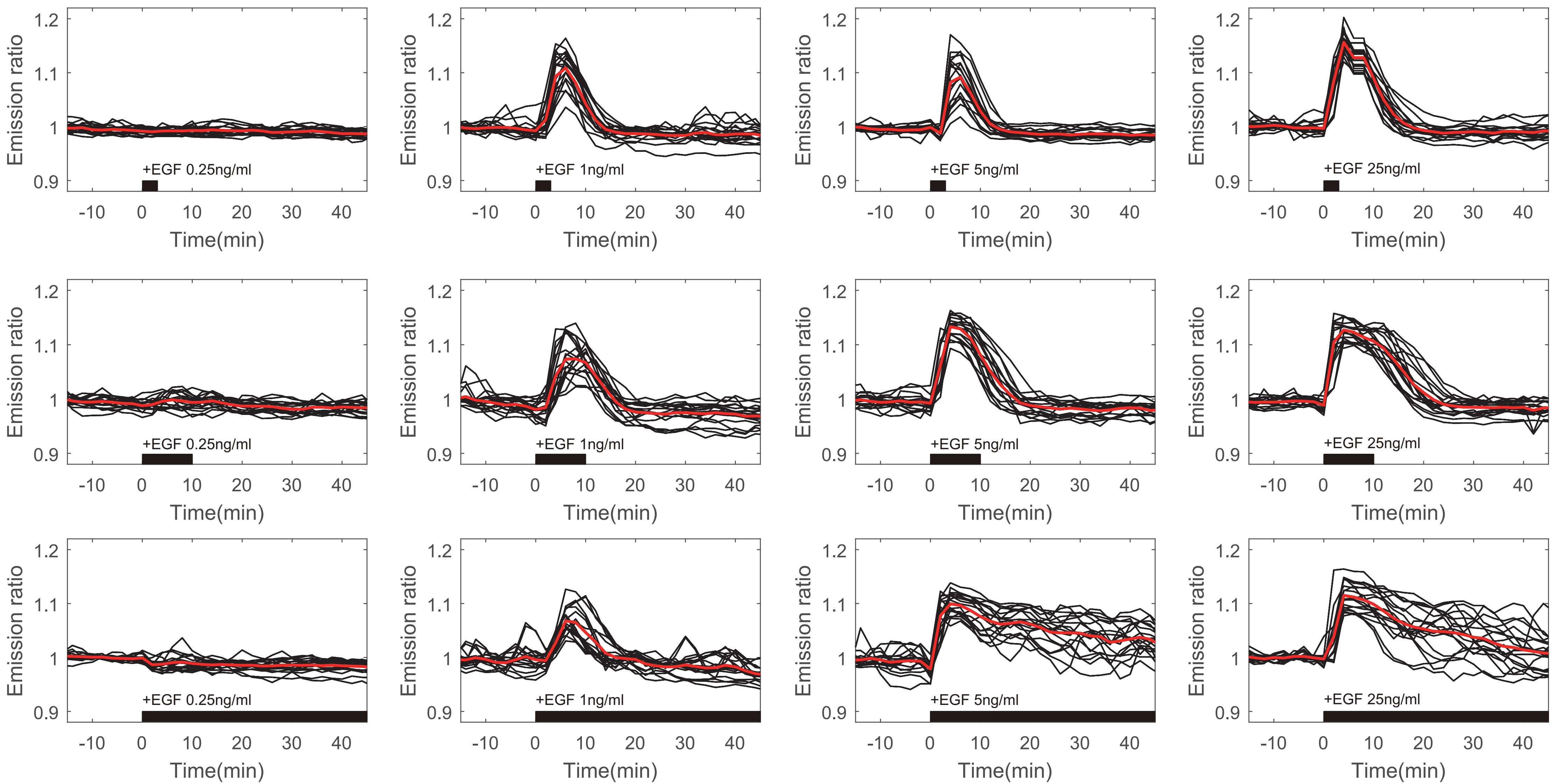

Emission ratio
Time(min)
+EGF 0.25ng/ml
+EGF 1ng/ml
+EGF 5ng/ml
+EGF 25ng/ml
